# Transplacental Transfer of Lumefantrine, Mefloquine, and Piperaquine: A Comparison of Concentrations in Mothers, Neonates, and Cord Blood

**DOI:** 10.1093/cid/ciaf552

**Published:** 2026-02-09

**Authors:** Makoto Saito, Mary Ellen Gilder, Verena Ilona Carrara, Laypaw Archusuksan, Hsa Eh, Naw Eh, Ma Ner, Aye Kyi Win, Laaongsri Niwetphongprai, Urairat Koesukwiwat, Joel Tarning, Nicholas J. White, François Nosten, Rose McGready

**Affiliations:** 1 Shoklo Malaria Research Unit, Mahidol-Oxford Tropical Medicine Research Unit, Faculty of Tropical Medicine, Mahidol University, Mae Ramat, Thailand; 2 Centre for Tropical Medicine and Global Health, Nuffield Department of Medicine, University of Oxford, Oxford, United Kingdom; 3 Division of Infectious Diseases, Advanced Clinical Research Center, Institute of Medical Science, University of Tokyo, Tokyo, Japan; 4 Faculty of Medicine, Institute of Global Health, University of Geneva, Geneva, Switzerland; 5 Mahidol Oxford Tropical Medicine Research Unit, Faculty of Tropical Medicine, Mahidol University, Bangkok, Thailand

**Keywords:** malaria in pregnancy, maternal-fetal drug transfer, transplacental passage, fetal drug exposure, Thailand-Myanmar border

## Abstract

**Background:**

Malaria in pregnancy causes adverse effects on the mother and fetus, requiring effective antimalarial treatment. Understanding the transplacental transfer of antimalarials is vital to assessing fetal safety and the risk of congenital malaria.

**Methods:**

We assessed a triad of blood samples (mother and neonatal capillary, cord) at delivery among women who were treated for uncomplicated malaria with artemether–lumefantrine (AL), artesunate–mefloquine (ASMQ), or dihydroartemisinin–piperaquine (DP) 4–8 weeks before delivery.

**Results:**

Antimalarial drug concentrations at delivery were measured in 90 women (25 AL, 29 ASMQ, 36 DP). Drug concentrations were detectable in neonates at birth at a maximum of 27, 42, and 55 days after the first dose of lumefantrine, mefloquine, and piperaquine, respectively. The blood concentrations were highest in the mother, followed by the neonate, and lowest in cord blood. Piperaquine showed the highest neonate-to-mother (N/M) ratio (geometric mean, 0.98; 95% confidence interval, 0.67–1.44; n = 32) followed by carboxy–mefloquine (0.90; 0.75–1.08; n = 27), desbutyl–lumefantrine (0.44; 0.30–0.65; n = 16), mefloquine (0.42; 0.38–0.47; n = 26), and lumefantrine (0.31; 0.07–1.36; n = 9). Higher maternal body mass index was associated with a lower N/M ratio of desbutyl–lumefantrine. Female neonatal sex and a longer interval following drug administration were associated with higher N/M ratios of carboxy–mefloquine. No increased risk of jaundice was observed.

**Conclusions:**

Antimalarial drugs crossed the placenta variably. Neonatal concentrations ranged from less than half (lumefantrine, mefloquine) to near maternal equivalence (piperaquine). Collection of neonatal capillary samples at birth should be considered in future studies.

Malaria in pregnancy causes adverse effects on both mother and fetus, with increased mortality in both [1]. This unfavorable impact on the fetus is prominent in placental malaria when the placenta is directly affected [2]. Falciparum malaria parasites can sequester in the placenta, causing mechanical obstruction of the microcirculation and alteration of placental pathophysiology, which lead to placental insufficiency and, consequently, intrauterine growth restriction [1, 2].

The placenta is an interface between the fetus and the mother that acts as an important organ between 2 separate blood circulatory systems: uteroplacental and fetoplacental. Many endogenous and external substances, including drugs, can permeate through the placenta, and some can be harmful or beneficial to the fetus [3]. Transplacental transfer of drugs can be mediated by either passive (or facilitated) diffusion following the gradient of drug concentrations or active transport by transporters expressed in the syncytiotrophoblasts. The magnitude of this transplacental transfer may vary, depending on the physicochemical properties of the compound [4].

The malaria parasite can cross the placenta through transmission of infected red blood cells [5], causing congenital malaria, with an estimated overall prevalence of 6.9% (95% confidence interval [CI], 4.8%–7.9%) in malaria-endemic areas [6]. Antimalarial drugs that cross the placenta can potentially prevent fetal infection by providing fetal preexposure prophylaxis. While it is known that some malaria antigens and immunoglobulin G against those antigens can be transferred to the fetus through the placenta [7, 8], the transplacental transfer of antimalarial drugs is an area that requires further exploration. While advocacy for the prevention of mother-to-child transmission of human immunodeficiency virus has led to significant research on transplacental transfer of antiretrovirals [9], evaluation of the efficacy (ie, prevention of congenital malaria) and safety of in utero fetal exposure to antimalarials to treat malaria, which is the most common parasitic disease in humans, remains a significant knowledge gap.

Here, we have compared the concentrations of 3 antimalarials, lumefantrine, mefloquine, and piperaquine, and their main metabolites among a triad sample set (maternal and neonatal capillary blood and cord blood) collected at delivery in pregnant women who were treated with these antimalarials in the weeks before delivery.

## Methods

### Study Design and Eligibility

This study was nested in a randomized, controlled trial (NCT01054248) conducted on the Thailand–Myanmar border between 2010 and 2016 when *Plasmodium falciparum* was decreasing rapidly and *Plasmodium vivax* became the main species affecting the study area. The detailed study designs and clinical and pregnancy primary outcomes have been reported elsewhere [10–12]. Briefly, women aged 18–45 years with confirmed pregnancy at any gestational age who had either asymptomatic or uncomplicated malaria by any species of malaria confirmed by microscopy were enrolled in the trial.

### Intervention

Three oral antimalarials were randomly allocated in a 1:1:1 ratio using sealed envelopes: extended-dose artemether–lumefantrine (AL+), artesunate–mefloquine (ASMQ), and dihydroarte-misinin–piperaquine (DP). AL+ was given as 5 tablets (20/120 mg artemether/lumefantrine per tablet) twice daily for 4 days with chocolate milk. The standard 3-day regimen was used for ASMQ and DP (Supplementary Material 1).

### Blood Sampling and Drug Concentration Measurement

Blood samples were collected at delivery if the women had received the study drugs within a specific time frame based on the half-lives of the antimalarial partner drugs: 31 days for AL+, 44 days for ASMQ, and 58 days for DP. Blood was collected in 4 sodium heparin hematocrit tubes (200 μL) from a finger prick (in mothers) or a heel prick (in neonates) or from the umbilical cord blood and centrifuged at 11 000 × g for 3 minutes. After centrifugation, plasma was taken and preserved in cryotubes at −80°C. High-performance liquid chromatography tandem mass spectrometry was used to measure the plasma concentration of antimalarials and their main metabolites, as described previously [13, 14].

### Statistical Analyses

Scatterplots with trend lines based on the fractional polynomial method were used to describe drug concentrations in cord and neonatal blood over time from the first dose to the collection of delivery samples. For each drug, 3 ratios (cord-to-mother [C/M], neonate-to-cord [N/C], and neonate-to-mother [N/M]) were calculated to assess the transplacental transfer. Geometric means were used to summarize them. The handling of observations below the lower limit of quantification (LLOQ) is described in Supplementary Material 2. Then, we determined whether the N/M ratio was associated with maternal or fetal characteristics or with the time interval between the last drug administration and the collection time of delivery samples [15] using a linear regression model with the robust variance estimator after log transformation. Samples collected before the elimination phase (4 AL+ sampled before completion of all doses, 2 ASMQ sampled within 24 hours of the last dose) and outliers identified by visual inspection were excluded from the models. To assess safety in the fetus, the Wilcoxon rank sum test was used to compare blood concentrations between neonates with jaundice and those without. Stata/MP 18.0 was used for statistical analyses.

### Ethics

All participants gave their written consent to participate after explanation in their language of choice. The parent randomized, controlled trial was approved by the Ethics Committee of the Faculty of Tropical Medicine, Mahidol University in Bangkok, and the Oxford Tropical Research Ethics Committee. The current data analysis was approved by the Research Ethics Review Committee of the Institute of Medical Science, University of Tokyo.

## Results

### Study Inclusion and Baseline Characteristics

Among 511 pregnant women enrolled, 137 delivered a live-born singleton within the timeframe after administration of the study drugs (Supplementary Figure 1). Among them, samples were collected at delivery in 94 women. After excluding samples with insufficient volume, neonate, cord, or both samples were available for further analyses in 90 women: 25 women in AL+ (17 for lumefantrine, 24 for desbutyl–lumefantrine), 29 in ASMQ (29 each for mefloquine and carboxy–mefloquine), and 36 in DP (36 for piperaquine). The discrepancy in the available numbers between lumefantrine and desbutyl–lume-fantrine was because only desbutyl–lumefantrine was above the LLOQ in some samples with insufficient volume.

Among 90 women assessed for antimalarial drug concentration at delivery, the mean age was 26.8 years, and 82% (74 of 90) had vivax malaria monoinfection (Table 1). The mean estimated gestational age was 35.7 weeks at the baseline administration of the study drug and 38.5 weeks at delivery. The time interval between the first dose and samples taken was, on average, 20.7 days (range, 0.6–55.1). In 84% (76 of 90) of the women, samples were collected within 60 minutes after delivery. The triad of samples was collected within 30 minutes of each other in 86% (77 of 90) of the women.

### Drug Concentrations in Cord and Neonate Over Time

**Figure 1:**
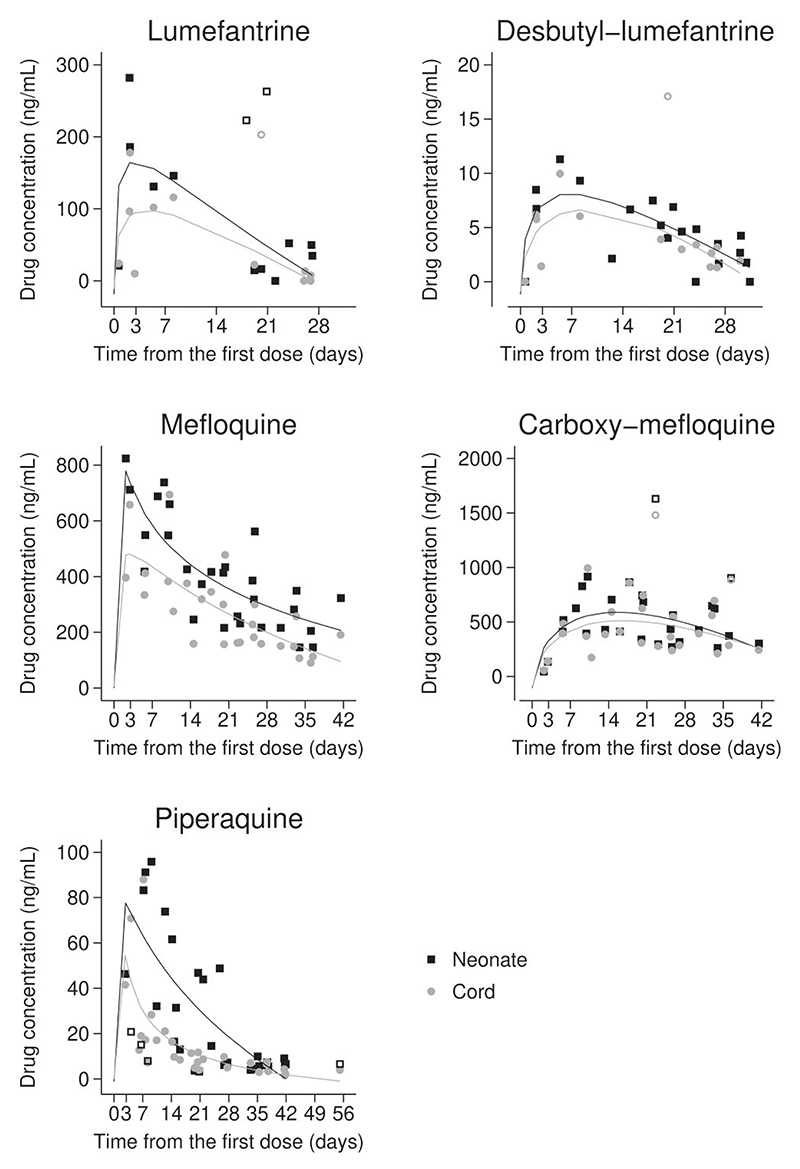
Antimalarial drug concentrations in neonate and cord blood samples over time. Trend lines based on fractional polynomial models are shown. Observations shown with hollow symbols were excluded from the trend lines as outliers.

Concentrations of the drugs at delivery over time since the first dose are shown in Figure 1. The maximum length of time from the first dose when drugs were still detectable in neonates and the observed concentrations on that day was 27 days for lumefantrine (34.8 ng/mL), 31 for desbutyl–lumefantrine (1.77 ng/mL), 42 for mefloquine (323 ng/mL) and carboxy–mefloquine (302 ng/mL), and 55 for piperaquine (6.61 ng/mL).

### C/M, N/C, and N/M Ratios

**Figure 2:**
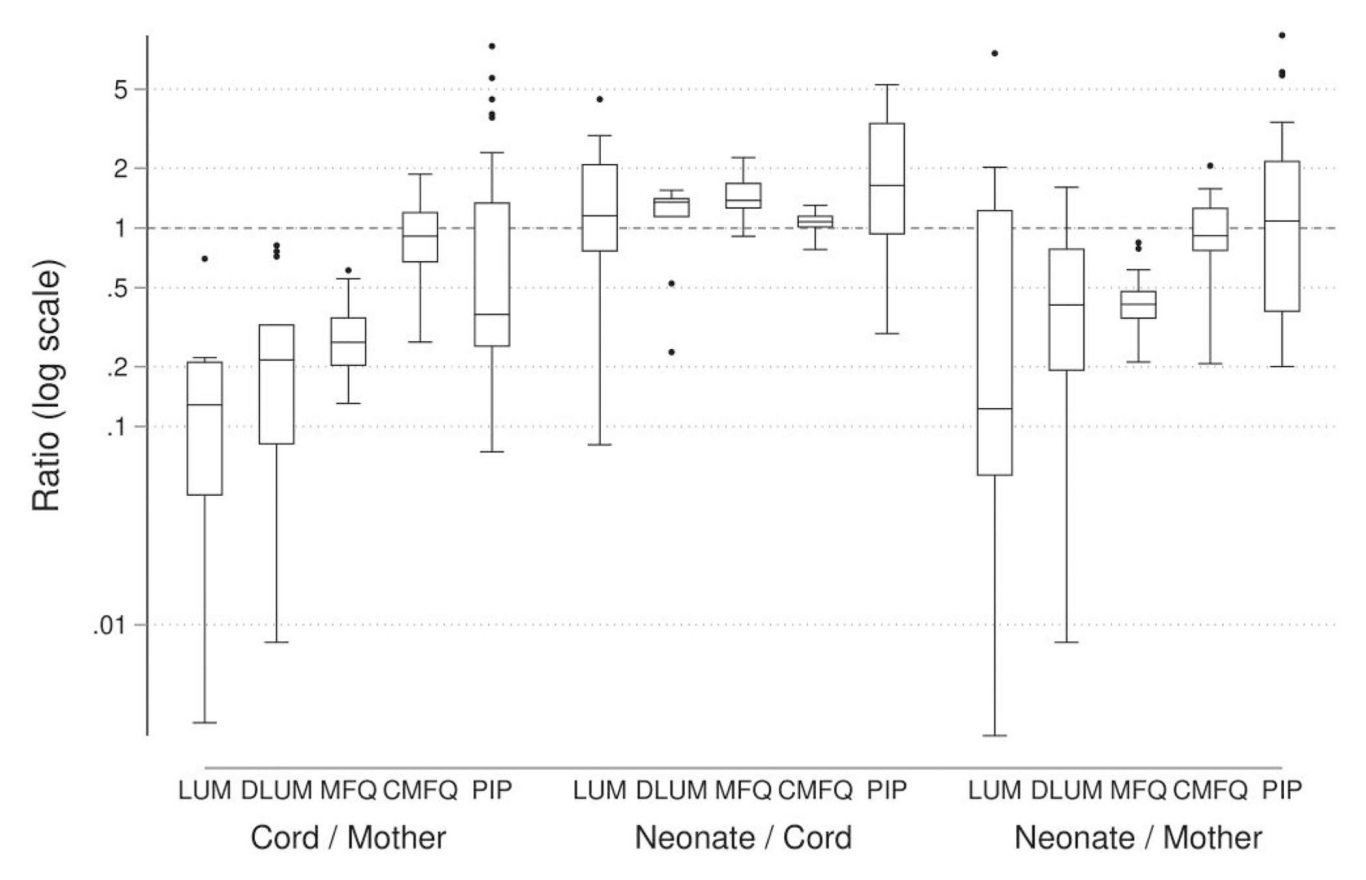
Distribution of the observed ratios for cord-to-mother, neonate-to-cord, and neonate-to-mother antimalarial drug concentrations. The ratios on the *y*-axis are presented on a log scale. The boxes represent the median values and interquartile ranges. Abbreviations: CMFQ, carboxy–mefloquine; DLUM, desbutyl–lumefantrine; LUM, lumefantrine; MFQ, mefloquine; PIP, piperaquine.

C/M, N/C, and N/M ratios were calculated for each paired measurement (Figure 2, Supplementary Figures 2–4), and geometric means and CIs were obtained after excluding the apparent outliers (Table 2). Drug concentrations in the cord and neonate were generally lower than those in the mother. The geometric mean of the C/M ratio was relatively low, ranging from 0.10 (95% CI, 0.06–0.18) for lumefantrine (n = 8) to 0.46 (95% CI, 0.30–0.72) for piperaquine (n = 27), except 0.83 (95% CI, 0.69–1.00) for carboxy–mefloquine (n = 26). Blood concentration was generally higher in neonates than in cords. The geometric mean of the N/C ratio ranged from 1.07 (95% CI, 1.02–1.12) for carboxy–mefloquine (n = 25) to 1.68 (95% CI, 1.28–2.21) for piperaquine (n = 26). The geometric mean of the N/M ratio was higher than the corresponding C/M ratio, ranging from 0.31 (95% CI, 0.07–1.36) for lumefantrine (n = 10) to 0.98 (95% CI, 0.67–1.44) for piperaquine (n = 32).

### Factors Associated With N/M Ratio

Maternal, fetal, and sampling factors associated with the N/M ratio were explored (Table 3). No characteristics, including smoking, were associated with the N/M ratio of lumefantrine (n = 8), mefloquine (n = 26), and piperaquine (n = 32). Higher maternal body mass index (BMI) was associated with a lower N/M ratio of desbutyl–lumefantrine (n = 15; 0.85-fold decrease in N/M ratio for each 1-kg/m^2^ increase in BMI; 95% CI, 0.74–0.97; *P* =.02; Supplementary Figure 5). Longer interval between the last drug administration and birth (delivery sample collection time), lower gravidity, and female neonatal sex were associated with an increased N/M ratio of carboxy–mefloquine (n = 25). Only longer time interval (adjusted ratio, 1.01; 95% CI, 1.00–1.02; *P* =.04) and female neonatal sex (adjusted ratio, 1.32; 95% CI, 1.00–1.72; *P* =.05) were independently associated with higher N/M ratio when they were assessed in the same model (Supplementary Figure 5).

### Congenital Malaria and Neonatal Jaundice

There was no congenital malaria. All neonatal blood samples (n = 88), cord blood samples (n = 75), and placenta smear samples (n = 76) were negative for malaria parasites by microscopy. After exclusion of 17 neonates with missing data on neonatal jaundice, 21.9% (16 of 73) had neonatal jaundice.

The median blood concentration was lower in neonates with jaundice than in neonates without jaundice for all compounds assessed, except mefloquine (median 482 ng/mL in the jaundice group and 349 ng/mL in the non-jaundice group), but none of the differences were statistically significant (Supplementary Table 1).

## Discussion

We demonstrated that antimalarials permeated the placenta and remained in the fetus for at least 4–8 weeks after maternal treatment against malaria. The ratios (C/M, N/C, N/M) were highly variable between different drugs, as expected. The drug concentration was highest in the mother, followed by the neonate, and lowest in cord blood. Although cord blood, instead of fetal blood, has been predominantly used to assess the transplacental transfer of drugs, few studies have directly compared cord and neonatal blood concentrations of any medications [9, 16, 17]. Our study indicates that the drug concentration in cord blood may underestimate the neonatal blood drug concentration. In our exploratory analysis, higher maternal BMI was associated with a lower desbutyl–lumefantrine N/M ratio, and female neonatal sex was associated with a higher carboxy–mefloquine N/M ratio.

Our study is one of the first clinical studies to assess the transplacental transfer of antimalarials, including chloroquine [18]. Although the safety of antimalarials in pregnancy has been a much-debated issue, only 1 study [19] has assessed partner drugs of artemisinin-based combination therapy, which is currently recommended for uncomplicated falciparum malaria, including in pregnant women [20]. The limited body of evidence related to the transplacental transfer of antimalarials is in stark contrast to that of antiretroviral drugs [9, 17].

A previous study that assessed transplacental transfer using a placenta perfusion model reported a C/M ratio of 0.75 ± 0.13 for mefloquine [19], which was higher than what we observed (geometric mean of 0.29 for C/M ratio and 0.42 for N/M ratio).

This difference between ex vivo placenta perfusion models and in vivo observations may be due to differences in protein binding between maternal and fetal blood [21]. It should be noted that in the case of malaria, the permeability of (treated) infected placentas in patients must differ from that of healthy placentas used in these ex vivo studies because both inflammation caused by infection and placental sequestration of the parasites can affect placental transfer [2].

C/M ratios were relatively low (<0.5) for all assessed antimalarials, except for carboxy–mefloquine (with a geometric mean of 0.83). All 3 antimalarials assessed in this study are lipophilic and relatively small molecules with molecular weights that range from 300 to 550 g/mol [22], which should theoretically facilitate the transplacental transfer by passive diffusion. Our observed low C/M ratios can be partly explained by their extremely high protein binding (>98%–99%) [9, 22]. Although protein binding generally decreases in pregnancy [23], lower albumin and alpha-1-acid glycoprotein levels in the fetus relative to those in the mother can lower the transplacental transfer of these highly protein-bound drugs [17].

Some variations in transplacental transfer between antimalarials can be explained by active transport that involves different transporters. P-glycoprotein (P-gp), a member of the ATP-binding cassette (ABC) transporter family, is an efflux transporter known to be expressed on the placenta [16], removing its substrates from the fetal circulation. While piperaquine is shown not to be a substrate of P-gp [24], lumefantrine is a substrate of P-gp [25, 26], which may be a reason for the lowest C/M and N/M ratios for lumefantrine among the assessed antimalarials. Mefloquine is an inhibitor of P-gp and other ABC transporters, and it can also act as a substrate [27–29]. Carboxy–mefloquine, the primary metabolite of mefloquine, is an agonist of the pregnane X receptor (PXR) [30], which suppresses the expression of the ABC transporters in the placenta in mice [31]. This suppression of the ABC transporters may lead to the increased C/M ratios of both mefloquine and carboxy–mefloquine compared with lumefantrine. Another animal study showed that PXR activation was blocked in male fetal rats [32], which may explain the lower N/M ratios of carboxy–mefloquine observed in male neonates in our study. Although the high blood concentration of carboxy–mefloquine in newborns (and presumably fetuses) does not lead to any beneficial effects on malaria as it is inactive against malaria parasites, induced suppression of ABC transporters may require caution for potential drug–drug interactions.

Another factor that affects the transplacental passage is metabolism by enzymes expressed in the placenta. However, this may not play a major role as all 3 antimalarials (and also artemisinin derivatives) are metabolized mainly by CYP3A4 [22, 33], which is not expressed in the placenta [16, 34]. Lumefantrine is reported to inhibit CYP2D6 [33], which is expressed in the placenta [16]. Although this does not affect the passage of lumefantrine, fetal exposure to the drugs metabolized by CYP2D6 can theoretically become higher when lumefantrine is co-administered.

Neonatal capillary blood concentrations were generally higher than cord blood concentrations. Although it has been assumed that drug concentrations are similar between cord and fetal blood, this assumption is supported by limited data, as most studies have used only cord blood samples [16, 35]. Indirect comparison among the studies on raltegravir, an antiretroviral drug, is consistent with our finding: neonatal blood concentration (N/M ratio 1.6~15, n = 6) [36, 37] was generally higher than cord blood concentration (C/M ratio 1~1.5) [9]. Hemoconcentration [38] and accumulation of antimalarials in red blood cells [22] can contribute to the higher concentrations in neonatal capillary blood compared with cord blood.

N/M ratios were larger than corresponding C/M ratios due to all of these factors, leading to N/C ratios greater than 1. In particular, piperaquine concentrations in neonates were close to those in mothers. This higher drug concentration in the fetus (neonate) could be beneficial in treating the fetus as well as in reducing the risk of congenital malaria by providing preexposure prophylaxis for neonates. Congenital malaria was rare in our study area but can be as high as 47% in some other malaria-endemic areas [6]. However, safety of the transferred drugs must be balanced against this potential benefit; we did not observe any increased risk of neonatal jaundice. The overall prevalence of neonatal jaundice (21.9%) among the women who were treated with antimalarials up to several weeks before their delivery was comparable to the background prevalence in this area (25%) [39], and the antimalarial drug concentrations were not higher in neonates with jaundice in the current study. Another potential safety concern includes the cardiotoxicity (QT prolongation) of antimalarials, particularly piperaquine. One previous study assessed QT intervals in neonates born to mothers who were chronically treated with hydroxychloroquine for autoimmune diseases. That study showed that maternal hydroxychloroquine use was not associated with an increased risk of QT prolongation in newborns and that drug concentrations in cord blood did not correlate with QT interval [40]. Although there is no direct evidence of cardiotoxicity of antimalarials other than chloroquine in neonates, we previously showed that the magnitude of QT prolongation was similar between chloroquine and piperaquine in pregnant women [12]. Nonetheless, caution is needed for intrauterine exposures to antimalarials if there are preexisting conditions that predispose to arrhythmia.

As C/M and N/M ratios were only measured at a single time point at delivery, they do not necessarily reflect the whole drug exposure in utero. However, C/M and N/M ratios are valuable sources of information for assessing placental transfer and drug exposure in utero, as it is not ethically possible to measure drug concentrations directly in cord blood or fetal blood before delivery. Amniocentesis is invasive and rarely available in malaria-endemic settings. Heel-prick blood collection is less invasive, technically easy, and widely used, including in malaria-endemic areas. Nonetheless, a sufficient volume of blood should be taken for measuring drug concentrations, particularly when the drug concentrations are low around LLOQ. We had to exclude some samples below the LLOQ that required dilution to measure drug concentrations, which could have overestimated the N/M ratio by selectively including those neonatal samples with higher concentrations.

Similarly, because we focused on transplacental transfer comparing mothers and neonates, factors that affect drug concentrations in mothers, including altered pharmacokinetics during pregnancy, were not discussed. Following previous research [15], we assessed the time since the last drug administration. However, this can be interpreted as a surrogate for maternal drug concentration, as maternal levels are largely determined by it during the elimination phase, which we analyzed. Although maternal characteristics, except for maternal BMI on desbutyl–lumefantrine, were not shown to be associated with N/M ratio, some other factors, such as maternal blood pressure or pH, may affect placenta permeability and thus should be explored in future studies. As physiologically based pharmacokinetic modeling techniques advance, they may produce more reliable predictions of fetal exposure at different gestational ages without invasive sampling, although confirmation of predictions with clinical data is required. Antimalarials from short-course treatments of 3–4 days were detected at a longer interval than expected. This may be due to some drugs being trapped and accumulating in fetal tissues via reabsorption from amniotic fluid [16] and can also be explained by the immature and slower fetal metabolism [41]. Future studies should consider assessing delivery samples among women who were treated earlier than the 4- to 8-week timeframe before delivery.

## Conclusions

Antimalarials can be detected in newborns up to 4–8 weeks after treatment of maternal infection. Piperaquine showed the highest N/M ratio, potentially reducing the risk of congenital malaria. Our results show that the C/M ratio, which is commonly used to assess the impact of transplacental transfer, may underestimate neonatal drug concentrations. While there is growing recognition to include pregnant women in drug studies, evidence for fetal exposure in utero remains rare. However, simple triad collection at delivery is possible and informative.

## Supplementary Material

Supplementary Data Supplementary materials are available at Clinical Infectious Diseases online. Consisting of data provided by the authors to benefit the reader, the posted materials are not copyedited and are the sole responsibility of the authors, so questions or comments should be addressed to the corresponding author.

Supplement

## Figures and Tables

**Table 1:** Baseline Characteristics of Women Assessed for Antimalarial Drug Concentration at Delivery and Delivery Sample Information

Characteristic	Lumefantrine (n = 25)^a^	Mefloquine (n = 29)	Piperaquine (n = 36)
Age, years	26.4 (7.3)	27.2 (6.4)	26.9 (6.7)
EGA at malaria treatment, weeks	35.2 (2.6)	36.1 (3.0)	35.6 (2.2)
Parity	1 [0–3]	1 [0–3]	1 [0–3.5]
Gravidity	2 [2–4]	3 [1–5]	3 [1–5]
Height, cm	149.6 (6.0)	149.8 (5.4)	150.4 (5.1)
Weight, kg	52.6 (6.4)	56.5 (6.7)	53.9 (7.7)
Body mass index, kg/m^2^	23.5 (2.6)	25.2 (2.8)	23.8 (3.0)
Malaria species			
Pf monoinfection	16% (4/25)	21% (6/29)	14% (5/36)
Pv monoinfection	84% (21/25)	76% (22/29)	86% (31/36)
Pf and Pv coinfection	0% (0/25)	3% (1/29)	0% (0/36)
Hematocrit at malaria episode, %	32.8 (3.6)	34.6 (4.4)	33.0 (3.9)
Smoking	40% (10/25)	21% (6/29)	25% (9/36)
Sample information			
EGA at delivery, weeks	37.8 (2.1)	38.8 (2.7)	38.7 (1.2)
Time from the first dose, days	22.1 (0.6–31.4)	20.2 (2.2–41.5)	20.7 (2.8–55.1)
Time from delivery to sample collection < 60 min	72% (18/25)	97% (28/29)	83% (30/36)

Information on the first date of study drug administration is presented. Mean (standard deviation), median (range) [interquartile range], or percentage (number) is shown. Abbreviations: EGA, estimated gestational age; Pf, *Plasmodium falciparum*; Pv, *Plasmodium vivax*.

a Both cord and neonatal samples were not available for lumefantrine (n = 8) or desbutyl–lumefantrine (n = 1).

**Table 2:** Geometric Mean and 95% Confidence Intervals of the Ratios of Antimalarial Drug Concentrations for Cord-to-Mother, Neonate-to-Cord, and Neonate-to-Mother

Analyte	Cord-to-Mother		Neonate-to-Cord		Neonate-to-Mother	
	n	Geometric Mean (95% CI)	n	Geometric Mean (95% CI)	n	Geometric Mean (95% CI)
Lumefantrine	8	0.10 (0.06–0.18)	7	1.43 (0.76–2.69)	9	0.31 (0.07–1.36)
Desbutyl–lumefantrine	12	0.21 (0.12–0.37)	9	1.22 (0.94–1.57)	16	0.44 (0.30–0.65)
Mefloquine	25	0.29 (0.25–0.34)	25	1.40 (1.28–1.53)	26	0.42 (0.38–0.47)
Carboxy–mefloquine	26	0.83 (0.69–1.00)	25	1.07 (1.02–1.12)	27	0.90 (0.75–1.08)
Piperaquine	27	0.46 (0.30–0.72)	26	1.68 (1.28–2.21)	32	0.98 (0.67–1.44)

Geometric mean and 95% CIs of the ratios are derived after log transformation, and back-transformed values are shown. Outliers based on Supplementary Figures 2–4 are excluded. Abbreviation: CI, confidence interval.

**Table 3:** Fetal, Maternal, and Sample Characteristics Associated With Neonate-to-Mother Ratios of Antimalarial Drug Concentrations

Variable	Lumefantrine			Desbutyl–Lumefantrine			Mefloquine			Carboxy–Mefloquine			Piperaquine		
	N	Coefficient (95% Cl)	*P* Value	N	Coefficient (95% Cl)	*P* Value	N	Coefficient (95% Cl)	*P* Value	N	Coefficient (95% Cl)	*P* Value	N	Coefficient (95% Cl)	P Value
Time since the last dose, days	8	1.12 (0.99–1.27)	.06	15	1.00 (0.94–1.06)	.98	26	1.00 (0.99–1.01)	.94	25	1.02 (1.01–1.02)	<.001	32	1.00 (0.97–1.03)	.93
EGA at delivery, weeks	8	1.03 (0.35–3.05)	.95	15	0.93 (0.71–1.22)	.57	26	0.93 (0.82–1.06)	.29	25	1.05 (0.91–1.20)	.49	32	1.24 (0.90–1.70)	.18
Age, years	8	1.06 (0.78–1.44)	.66	15	1.02 (0.98–1.07)	.33	26	1.00 (0.98–1.02)	.90	25	0.99 (0.97–1.01)	.40	32	1.01 (0.96–1.06)	.79
Gravidity 1	1	0.15 (0.01–1.65)	.10	3	0.70 (0.22–2.23)	.51	9	0.98 (0.73–1.31)	.86	9	1.39 (1.05–1.83)	.02	11	1.16 (0.49–2.74)	.73
2	3	1.59 (0.01–213.76)	.82	5	0.83 (0.31–2.25)	.70	5	0.97 (0.77–1.22)	.78	4	1.14 (0.86–1.51)	.35	4	0.60 (0.18–2.02)	.39
≥3	4	Reference		7	Reference		12	Reference		12	Reference		17	Reference	
Height, cm	8	1.17 (0.91–1.52)	.18	15	1.05 (0.98–1.11)	.14	26	0.99 (0.97–1.01)	.26	25	0.99 (0.96–1.02)	.45	32	0.99 (0.93–1.06)	.81
Weight, kg	8	0.85 (0.61–1.17)	.26	15	0.97 (0.91–1.03)	.32	26	0.99 (0.98–1.01)	.54	25	1.01 (0.99–1.03)	.56	32	1.02 (0.98–1.07)	.31
Body mass index, kg/m^2^	8	0.55 (0.28–1.08)	.08	15	0.85 (0.74–0.97)	.02	26	1.00 (0.95–1.05)	.95	25	1.03 (0.98–1.08)	.27	32	1.09 (0.94–1.25)	.25
Smoking	3	3.23 (0.07–158.44)	.49	6	1.28 (0.51–3.25)	.57	5	0.99 (0.78–1.26)	.95	4	1.05 (0.74–1.50)	.76	8	0.86 (0.35–2.12)	.73
No	5	Reference		9	Reference		21	Reference		21	Reference		24	Reference	
Neonatal sex: Male	2	Reference		7	Reference		16	Reference		16	Reference		14	Reference	
Female	6	0.23 (0.02–2.45)	.18	8	0.75 (0.34–1.68)	.45	10	1.04 (0.81–1.34)	.73	9	1.42 (1.10–1.83)	.01	18	0.81 (0.37–1.80)	.60
*z*score of birth weight for EGA	6	2.71 (0.14–51.42)	.40	13	1.13 (0.62–2.07)	.66	26	1.07 (0.85–1.34)	.54	25	0.95 (0.74–1.22)	.70	32	1.33 (0.86–2.06)	.19

Univariable linear regression with the robust variance estimator was conducted after log transformation of neonate-to-mother ratios, and exponentiated coefficient and 95% CIs are shown. Neonate-to-mother ratio will increase by the factor of the values shown as coefficient for every 1 unit increase in the dependent variable. Outliers based on Supplementary Figures 2–4, and samples collected before the elimination phase are excluded. Abbreviations: CI, confidence interval; EGA, estimated gestational age.

## Data Availability

Data are available from MORU Tropical Health Network upon request at the following link: https://www.tropmedres.ac/units/moru-bangkok/bioethics-engagement/data-sharing.
