# Supplementary material for: Transplacental Transfer of Lumefantrine, Mefloquine, and Piperaquine: A Comparison of Concentrations in Mothers, Neonates, and Cord Blood
Source: Clin Infect Dis. Author manuscript; Available in PMC 2026 Jan 31. (PMC7618698; doi:10.1093/cid/ciaf552)
Supplement: Supplement [file EMS212263-supplement-Supplement.pdf]

Transplacental transfer of lumefantrine, mefloquine and piperaquine: a comparison of concentrations in mothers, neonates and cord blood

Supplement 1. Dosing Tables of artesunate-mefloquine and dihydroartemisinin-piperaquine

Artesunate-mefloquine

Fixed Dose: artesunate / mefloquine hydrochloride (100 mg / 220 mg per tablet, Far-Manguinhos, Brazil)

Weight (kg)	Number of tablets
>29	2

Loose dose: artesunate (50 mg per tablet, Guilin, China) and mefloquine (250 mg per tablet, Atlantic Laboratories Corp, Thailand)

Mefloquine	
Weight (kg)	Number of tablets
30-35	1
36-42	1.25
43-51	1.5
52-57	1.75
58-67	2
68-73	2.25
74-82	2.5
83-89	2.75
90-100	3

Artesunate	
Weight (kg)	Number of tablets
30-32	2.5
33-34	2.75
35-39	3
40-42	3.25
43-45	3.5
46-48	3.75
49-51	4
52-54	4.25
55-57	4.5
58-60	4.75
61-64	5
65-67	5.25
68-70	5.5
71-73	5.75
74-76	6
77-79	6.25

Dihydroartemisinin-piperaquine: dihydroartemisinin/piperaquine (40 mg/320 mg per tablet, Holley Pharmacy, China)

Weight (kg)	Number of tablets
31-40	2
41-50	2.5
51-60	3
61-70	3.5
71-84	4
85-100	5

Supplement 2. Handling of data below lower limit of quantification

The lower limit of quantification (LLOQ) was 7.77 ng/mL for lumefantrine, 0.808 ng/mL for desbutyl-lumefantrine, 7.64 ng/mL for mefloquine, 7.64 ng/mL for carboxy-mefloquine, and 1.20 ng/mL for piperaquine.

To calculate cord-to-mother, neonate-to-cord and neonate-to-mother ratios, observations below LLOQ were replaced with half of the LLOQ values unless both the denominator and numerator were lower than LLOQ to alleviate the bias by selectively excluding observations with lower drug concentrations. Dilution was required to measure drug concentrations in low-volume samples. If drug concentrations in diluted samples fell below LLOQ, the sample was excluded from analysis because the raised LLOQ decreases the precision of the measurement.

Table S1. Comparison of drug concentrations in neonatal blood between neonates with and without jaundice.

Compound	Drug concentration (median, range) in neonates (ng/ml)		p-value
	Neonatal jaundice	No jaundice	
Lumefantrine	74 (16.4-131) (n=2)	166 (0-282) (n=8)	0.53
Desbutyl-lumefantrine	3.4 (0-11.3) (n=4)	5.2 (0-9.3) (n=13)	0.55
Mefloquine	482 (146-824) (n=4)	349 (146-738) (n=23)	0.49
Carboxy-mefloquine	429 (44.2-904) (n=4)	429 (135-1630) (n=23)	0.72
Piperaquine	7.4 (3.3-20.8) (n=6)	31.8 (4.6-95.9) (n=18)	0.07
p-value by exact Wilcoxon's rank sum test.			

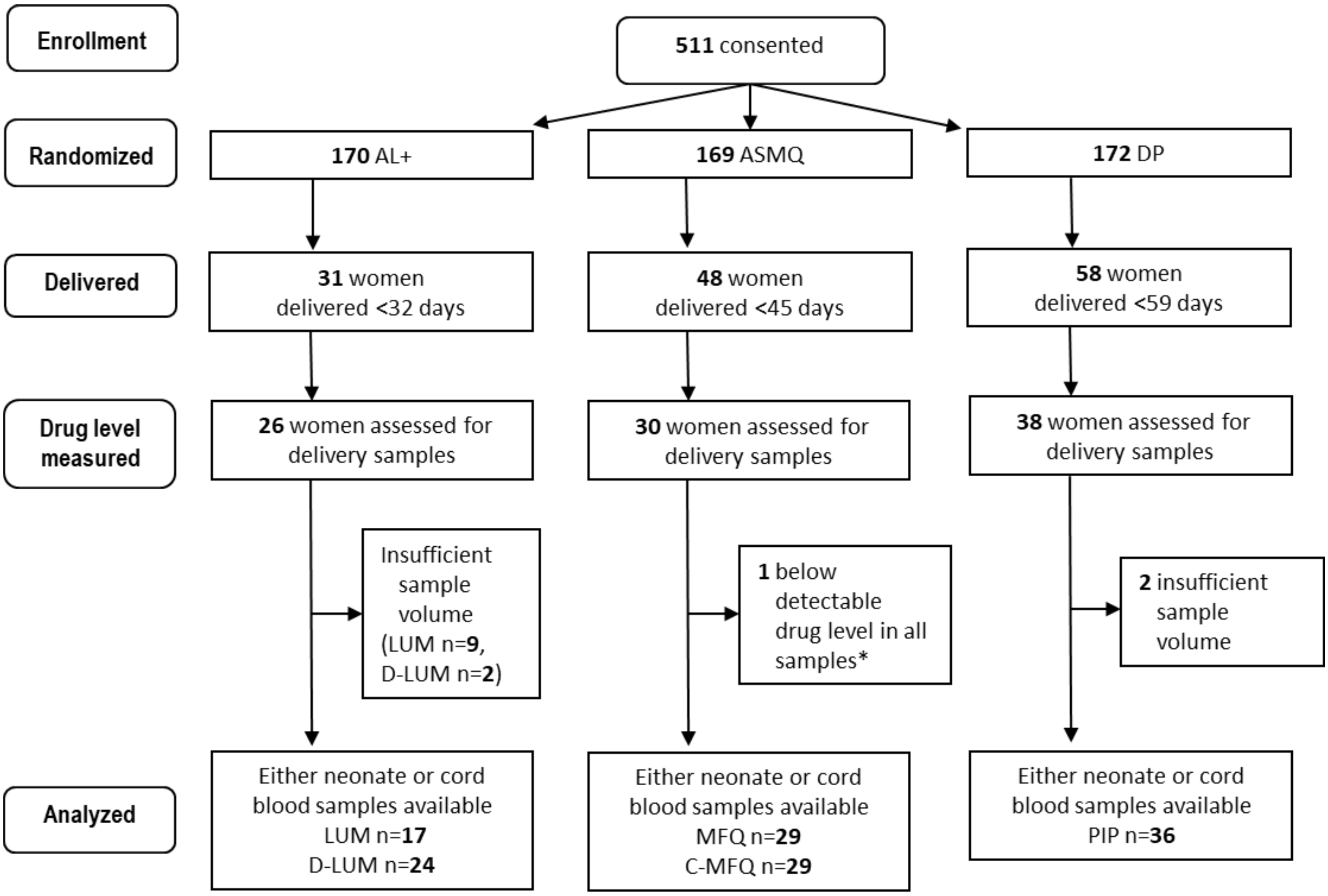

Figure S1. Study flowchart.

* All the samples were taken within 1 hour after the first dose.

AL+: extended regimen Artemether-lumefantrine, ASMQ: artesunate-mefloquine, CMFQ: carboxy-mefloquine, DLUM: desbutyl-lumefantrine, DP: dihydroartemisinin-piperaquine, LUM: lumefantrine, MFQ: mefloquine, PIP: piperaquine

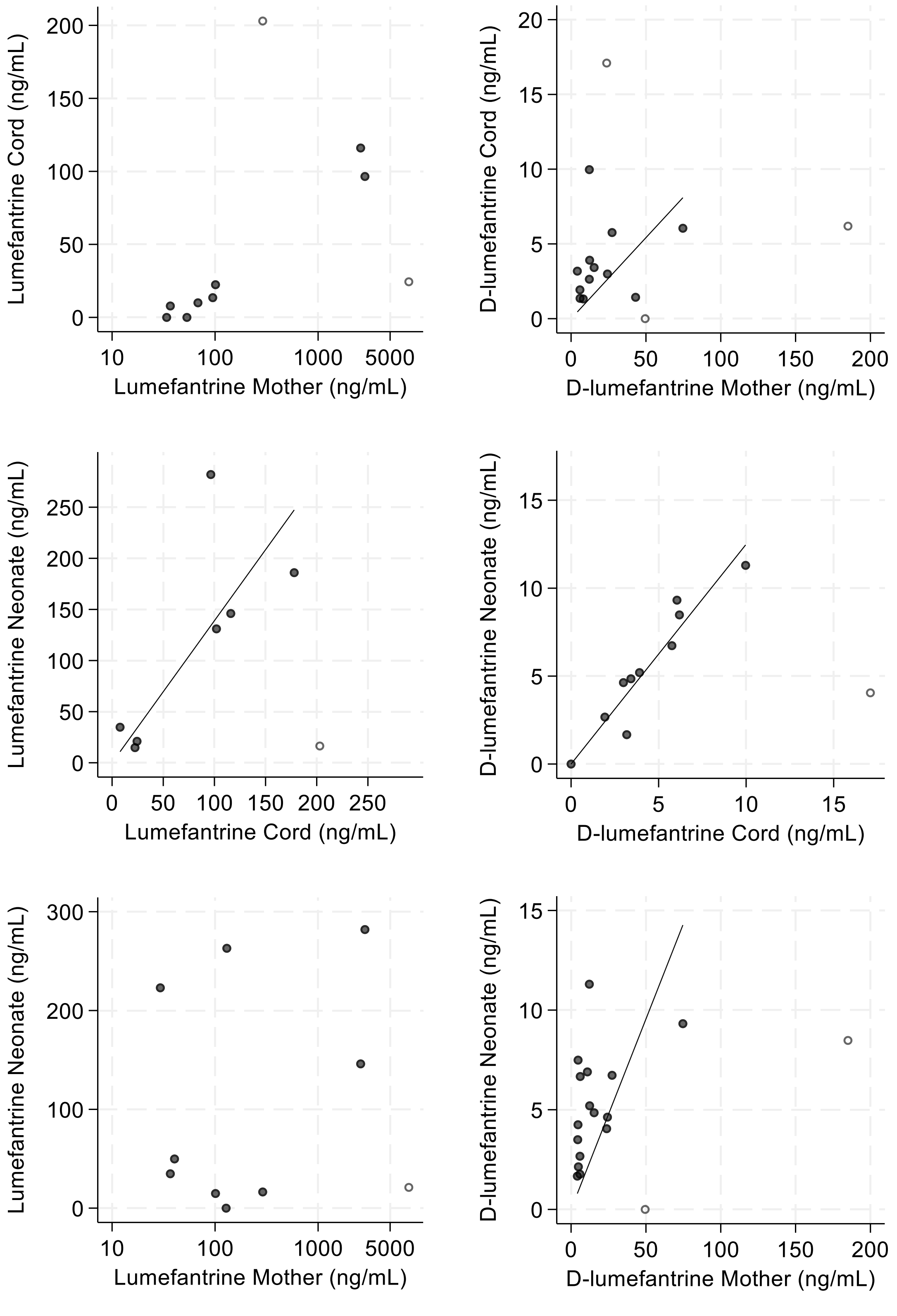

Figure S2. Concentrations of lumefantrine and desbutyl-lumefantrine (D-lumefantrine) in paired maternal, neonatal and cord blood samples. Hollow circles were excluded from models as outliers.

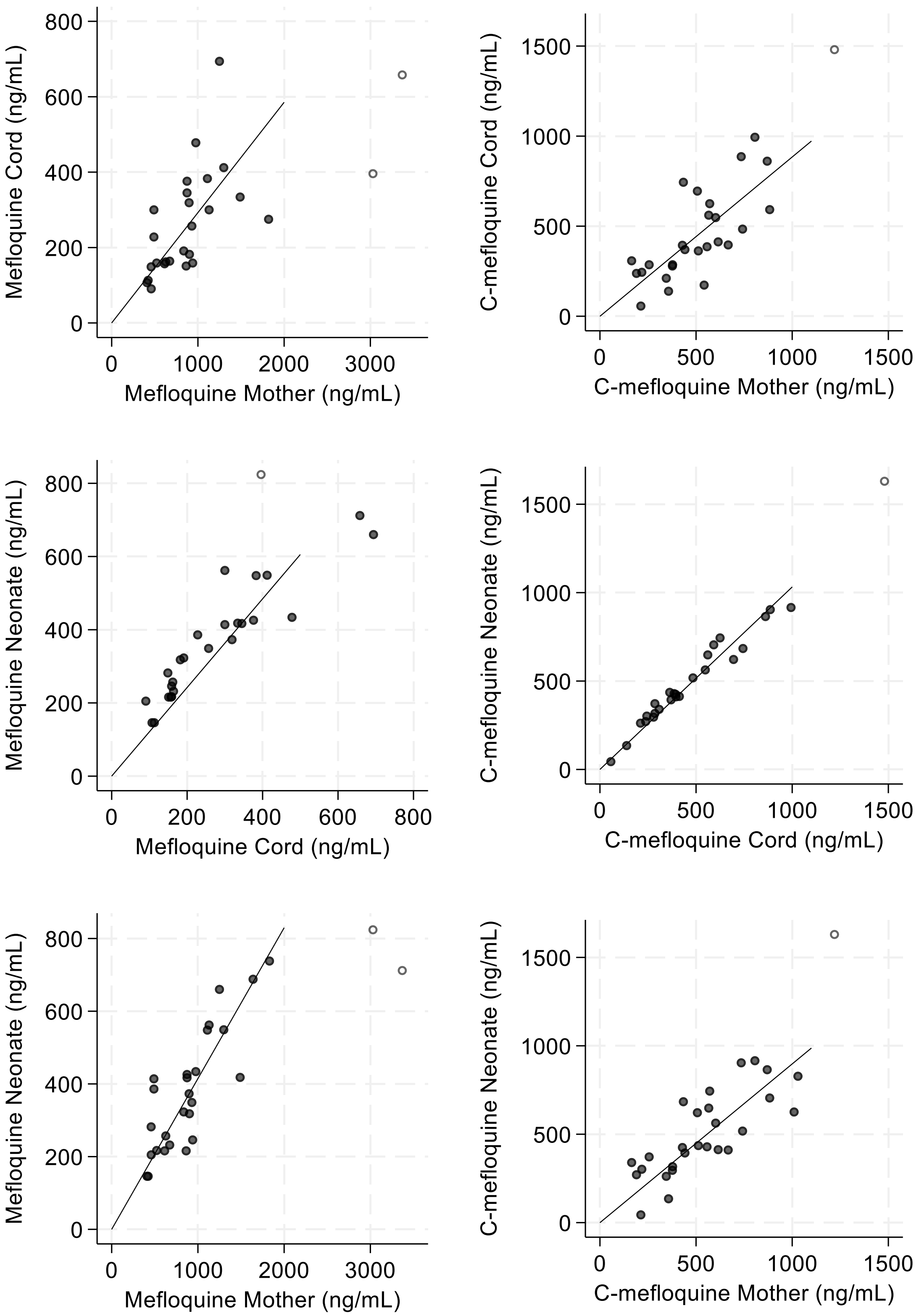

Figure S3. Concentrations of mefloquine and carboxy-mefloquine (C-mefloquine) in paired maternal, neonatal and cord blood samples. Hollow circles were excluded from models as outliers.

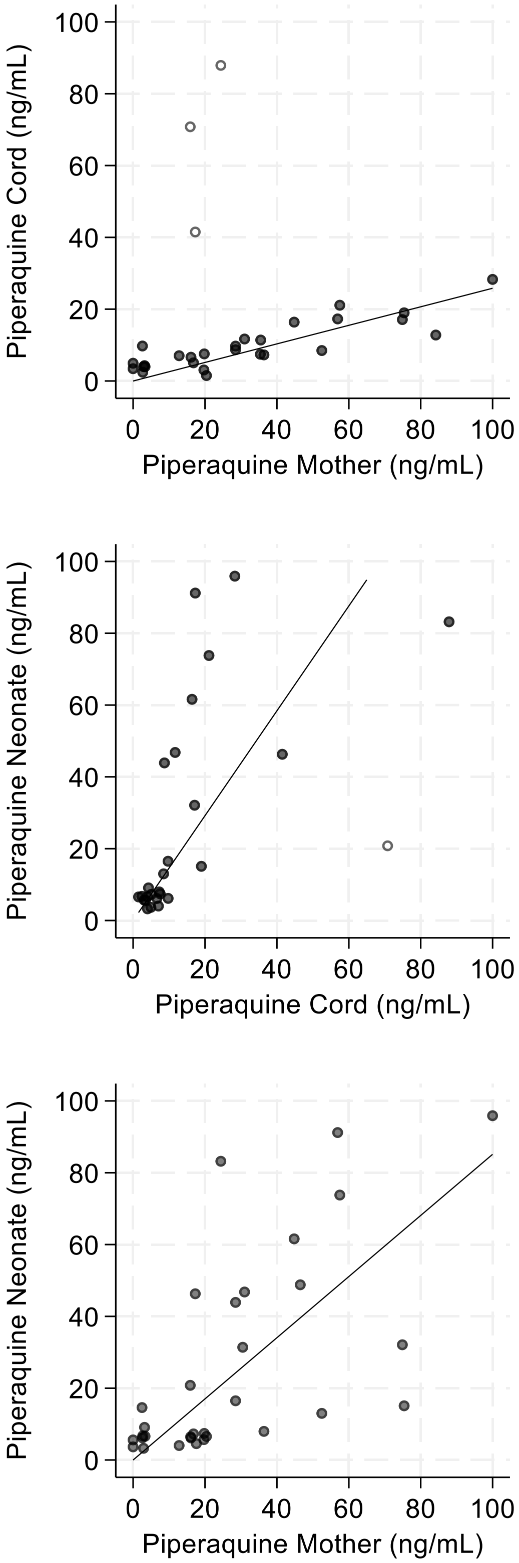

Figure S4. Concentrations of piperaquine in paired maternal, neonatal and cord blood samples. Hollow circles were excluded from models as outliers.

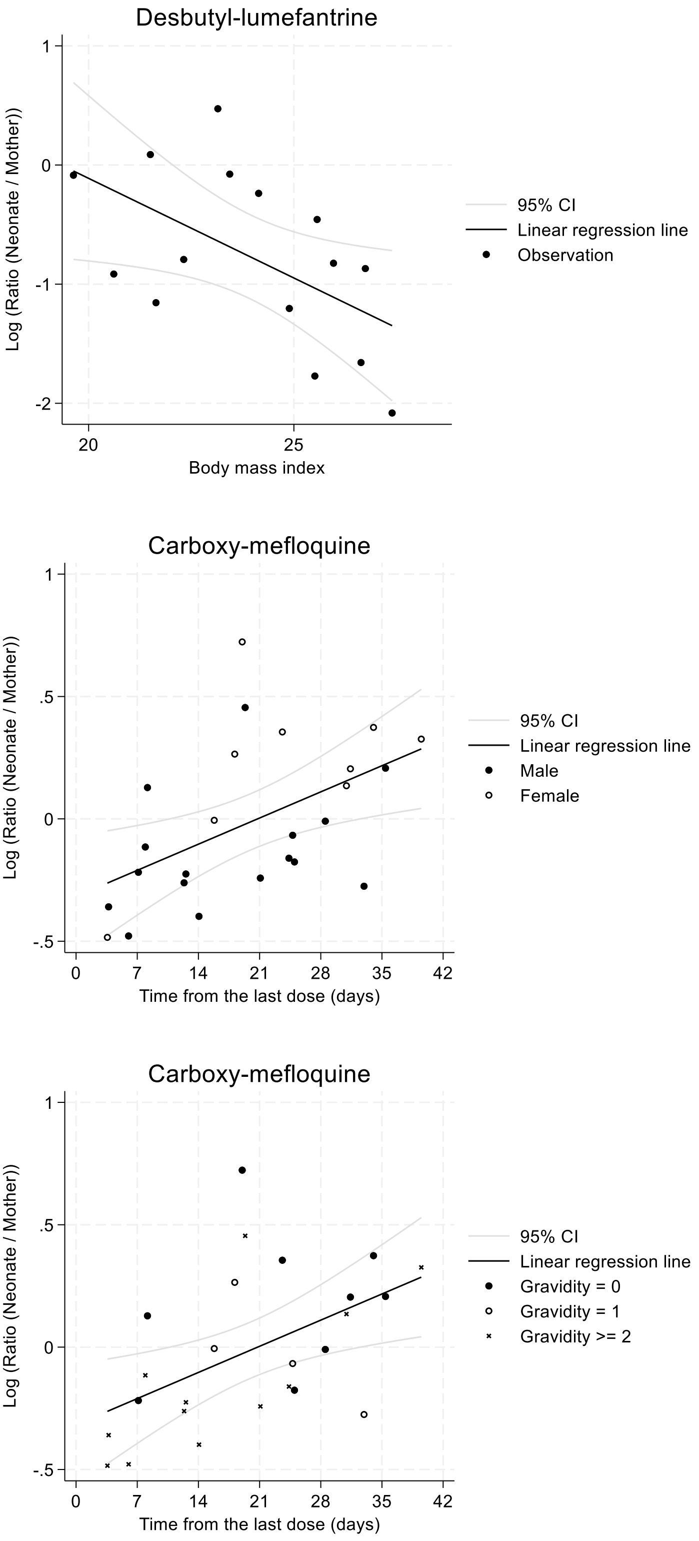

Figure S5. Scatter plots of neonate-to-mother ratio and associated factors for desbutyl-lumefantrine and carboxy-mefloquine. Observed values are plotted with a linear prediction line with 95% confidence intervals (CI).
